# Docking of THPDTPI: to explore P-selectin as a common target of anti-tumor, anti-thrombotic and anti-inflammatory agent

**DOI:** 10.18632/oncotarget.19374

**Published:** 2017-07-19

**Authors:** Haimei Zhu, Yuji Wang, Ce Song, Qiqi Feng, Jianhui Wu, Shurui Zhao, Lin Gui, Xiaoyi Zhang, Ming Zhao, Shiqi Peng

**Affiliations:** ^1^ College of Pharmaceutical Sciences of Capital Medical University, Beijing, China; ^2^ Beijing Area Major Laboratory of Peptide and Small Molecular Drugs, Beijing, China; ^3^ Engineering Research Center of Endogenous Prophylactic of Ministry of Education of China, Beijing, China; ^4^ Beijing Laboratory of Biomedical Materials, Beijing, China; ^5^ Department of Biomedical Science and Environmental Biology, Kaohsiung Medical University, Kaohsiung, Taiwan; ^6^ Guangxi Pusen Biotechnology Co. Ltd., Guilin, China

**Keywords:** P-selectin, anti-tumor, anti-thrombosis, anti-inflammation, target

## Abstract

The impact of soluble P-selectin on tumor growth, thrombosis and inflammation has been individually documented. Whether the down-regulation of P-selectin expression can simultaneously slow the tumor growth, inhibit the thrombosis and attenuate the inflammatory response remains unknown. In this context, (2′S,5′S)- tetrahydropyrazino[1’,2’:1,6]-di{2,3,4,9-tetrahydro-1*H*-pyrido[3,4-b]indole}-1’,4’-dione (THPDTPI) was designed as an inhibitor of P-selectin. The suitable docking of THPDTPI towards the active site of P-selectin, the significant down-regulation of THPDTPI to P-selectin expression, and the direct action of THPDTPI on P-selectin suggest that P-selectin could be a target of THPDTPI. *In vivo* THPDTPI possesses the anti-tumor activity, the anti-thrombotic activity and the anti-inflammatory activity. This implies that targeting P-selectin is of essential importance for this triple activity. The minimal effective doses of THPDTPI inhibiting the tumor growth, the rat arterial thrombosis and the mouse ear edema are 0.01 μmol/kg, 0.1 μmol/kg and 0.001 μmol/kg, respectively. Atomic force microscopy images and FT-MS spectra showed that the adhesion of THPDTPI onto the surfaces of the platelets may be the first step of P-selectin targeting. Besides, the dependence of the triple action of THPDTPI inhibiting the tumor growth, the thrombosis and the inflammation on the decrease of the soluble P-selectin led to the correlation of the soluble P-selectin with the serum TNF-α and serum IL-8.

## INTRODUCTION

As an adhesion receptor P-selectin is stored in α-granules of the resting platelets, may be expressed by the activated platelets, and becomes a soluble form (sP-selectin). The impact of sP-selectin on the cancer, the thrombosis and the inflammation has been individually investigated. The relevant rise of sP-selectin was associated with the enhancement of tumor growth and tumor metastasis [1, 2]. The obvious increase of the serum level of sP-selectin was considered a risk of developing thrombotic diseases [3–5]. High concentration of sP-selectin in blood circulation was correlated with the attack of inflammatory diseases [6, 7]. However, whether the down-regulation of P-selectin expression can simultaneously slow the tumor growth, inhibit the thrombosis and attenuate the inflammatory response remains unknown. In this context, we hypothesized that an inhibitor of P-selectin could simultaneously exhibit anti-tumor, anti-thrombotic and anti-inflammatory activities. Targeting the active site of P-selectin we computationally screened the anti-thrombotic tetrahydro-β-carbolines and the anti-tumor β-carbolines in our sample library. This led to the assignment of (2′S,5′S)-tetrahydropyrazino[1′,2′:1,6]-di{2,3,4,9-tetrahydro-1*H*-pyrido[3,4-b]indole}-1′,4′-dione (THPDTPI), a previously reported anti-tumor agent [8], as a potential inhibitor of P-selectin.

## RESULTS

### THPDTPI been fit for the active site of P-selectin

**Figure 1:**
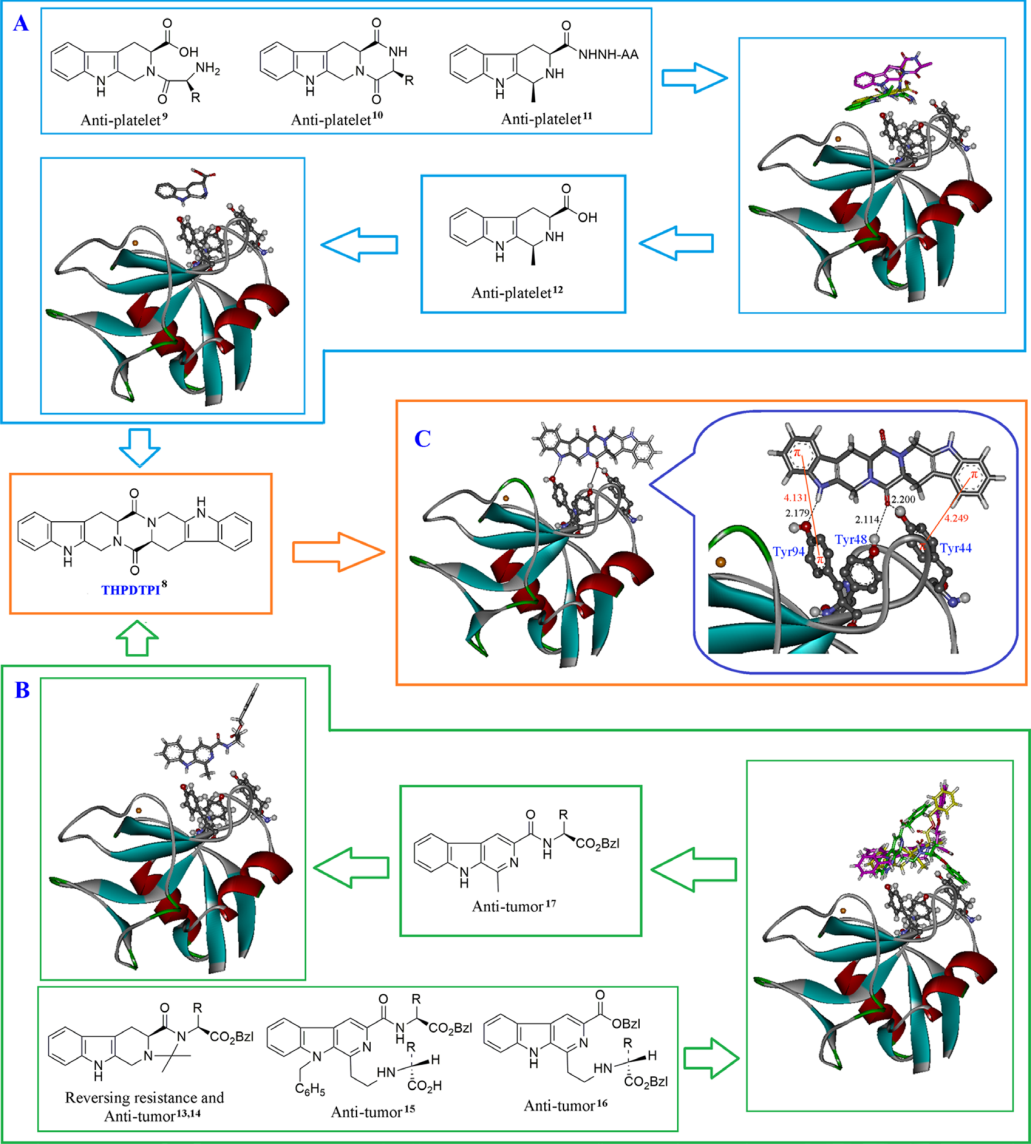
Docking investigation (**A**) Docking of 4 classes of anti-thrombotic tetrahydro-β-carbolines from our sample library into the active site of P-selectin; (**B**) Docking of 3 classes of anti-tumor β-carbolines from our sample library into the active site of P-selectin; (**C**) THPDTPI is identified as a potential inhibitor of P-selectin: THPDTPI binds Tyr residue rich area of the active pocket and thereby leads to forming 3 hydrogen bonds, i.e. the hydrogen bond of pyrole H of THPDTPI with the hydroxyl oxygen of Tyr94 residue, and two hydrogen bonds of carbonyl oxygen of THPDTPI with the hydroxyl hydrogen of Tyr48 residue and the hydroxyl hydrogen of Tyr44 residue. Besides, the short distance between the benzene rings of THPDTPI with the phenolic rings leads to the 2 π-π stacking, thereby enhances the formation of the hydrogen bonds. These interactions lead the docking free energy of THPDTPI to be –12.29 kcal/mol, which is a lower value than those of the 7 classes of β-carbolines.

The structural analysis and the comparison of the docking scores of 4 classes of anti-platelet tetrahydro-β-carbolines of our sample library were performed [9–11], and this led to the assignment of P-selectin inhibitor, 1-methyl-tetrahydro-β-carboline-3-carboxilic acid [12] (Figure 1A). The structural analysis and the comparison of the docking scores of 3 classes of anti-tumor β-carbolines of our sample library were also performed [13–16], and this led to the assignment of P-selectin inhibitor, 1-methyl-β-carboline3-carboxyl-amino acid benzyl esters [17] (Figure 1B). The structural analysis and the comparison of the docking scores of 1-methyl-tetrahydro-β-carboline-3-carboxilic acid and 1-methyl-β-carboline-3-carboxyl-amino acid benzyl esters led to the assignment of THPDTPI as a potential inhibitor of P-selectin. Figure 1C indicates that THPDTPI can properly dock into P-selectin and form the essential hydrogen bonds and π-π interactions with the side chains of the residues Tyr44, Tyr48 and Tyr94 of the active site of the average structure of P-selectin [18].

### AFM images visualize the adhesion of THPDTPI on platelet surfaces

**Figure 2:**
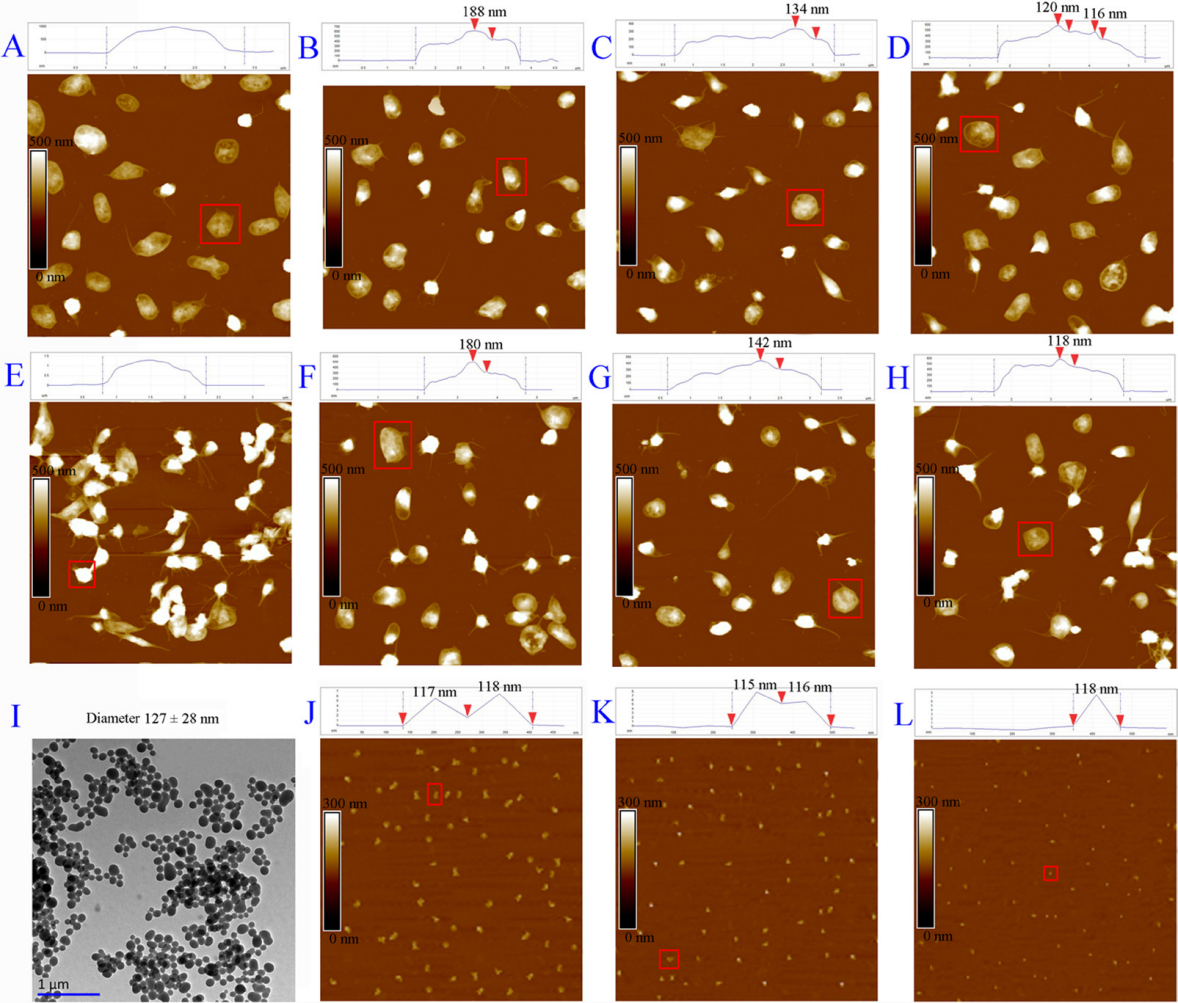
AFM imaged adhesion of THPDTPI’s nanoparticles on the surfaces of rat platelets (**A**) AFM image of resting rat platelets; (**B**) AFM image of resting rat platelets with THPDTPI (5 × 10^−8^ M); (**C**) AFM image of resting rat platelets with THPDTPI (5 × 10^−9^ M); (**D**) AFM image of resting rat platelets with THPDTPI (5 × 10^−10^ M); (**E**) AFM image of AA activated rat platelets; (**F**) AFM image of AA activated rat platelets with THPDTPI (5 × 10^−8^ M); (**G**) AFM image of AA activated rat platelets with THPDTPI (5 × 10^−9^ M); (**H**) AFM image of AA activated rat platelets with THPDTPI (5 × 10^−10^ M); (**I**) TEM image of THPDTPI (5 × 10^−8^ M), which was recorded as described in the literature [8]; (**J**) AFM image of THPDTPI in NS (5 × 10^−8^ M); (**K**) AFM image of THPDTPI in NS (5 × 10^−9^ M); (**L**) AFM image of THPDTPI in NS (5 × 10^−10^ M).

We considered that to target P-selectin THPDTPI should firstly adhere on the surfaces of the platelets. To visualize the adhesion, the atomic force microscopy (AFM) images of THPDTPI, resting platelets, arachidonic acid (AA) activated platelets, resting platelets with THPDTPI and AA activated platelets with THPDTPI were recorded according to the literature method [19]. Figure 2A is the AFM image of the resting platelets, and shows that the resting platelets have smooth surface without pseudopodia. Figure 2B, 2C and 2D are the AFM images of the resting platelets treated with THPDTPI. On the surfaces of the resting platelets treated with 5 × 10^−8^, 5 × 10^−9^ and 5 × 10^−10^ M of THPDTPI, there are nanoparticles of 188 nm, 134 nm and 120 nm in height, respectively. In this case the resting platelets still have no pseudopodia, and the height of the nanoparticles of THPDTPI concentration-dependently reduces. Figure 2E is the AFM image of AA activated platelets, and shows that these platelets have smooth surface, extend pseudopodia and form aggregators. Figure 2F, 2G and 2H are the AFM images of AA activated platelets treated with THPDTPI. On the surfaces of AA activated platelets treated with 5 × 10^−8^, 5 × 10^−9^ and 5 × 10^−10^ M of THPDTPI, there are nanoparticles of 188 nm, 142 nm and 118 nm in height, respectively. THPDTPI concentration-dependently inhibits AA activated platelets to extend pseudopodia, and completely inhibits AA activated platelets to form aggregator. Similarly, the height of the nanoparticles of THPDTPI concentration-dependently reduces.

To explore the difference between the adhesion of THPDTPI with the resting platelets and AA activated platelets, a FT-MS analysis was performed. Briefly, the resting platelets and AA activated platelets incubated with 5 × 10^−8^ M THPDTPI were sufficiently washed with ultrapure water, centrifuged at 500 g for 10 min, received ultrasonication in methanol for 10 min, centrifuged at 500 g for 10 min and the methanol extracts were analyzed with FT-MS. It was found that the methanol extract of AA activated platelets, but not the methanol extract of the resting platelets, gave a negative ion peak at 431.13315, the mass of THPDTPI plus Cl. Therefore only the adhesion of THPDTPI with AA activated platelets is stable enough. This difference could be attributed to P-selectin is stored in α-granules of the resting platelets but occurs on the surface of AA activated platelets. The FT-MS spectrograms are provided as Supplementary Figure 1 and Supplementary Figure 2 of Supporting Information.

Figure 2J, 2K and 2L are the AFM images of THPDTPI in NS. The height of the nanoparticles of 5 × 10^−8^ M, 5 × 10^−9^ M and 5 × 10^−10^ M THPDTPI are 115–118 nm. Figure 2I is the transmission electron microscopy (TEM) image of THPDTPI in water (5 × 10^−8^ M), and the diameter of the particles is 127 ± 28 nm.

To confirm these observations, the *in vitro* anti-platelet aggregation activity assay was performed by following a standard method. It was found that THPDTPI was capable of effectively inhibiting the platelet aggregation induced by adenosine diphosphate (ADP), and the IC_50_ value of THPDTPI against the platelet aggregation was 0.26 nM. The *in vitro* anti-platelet aggregation activity assay of THPDTPI was described in the Supplementary Materials.

### FT-MS spectra evidence the adhesion of THPDTPI on platelet surfaces

**Figure 3:**
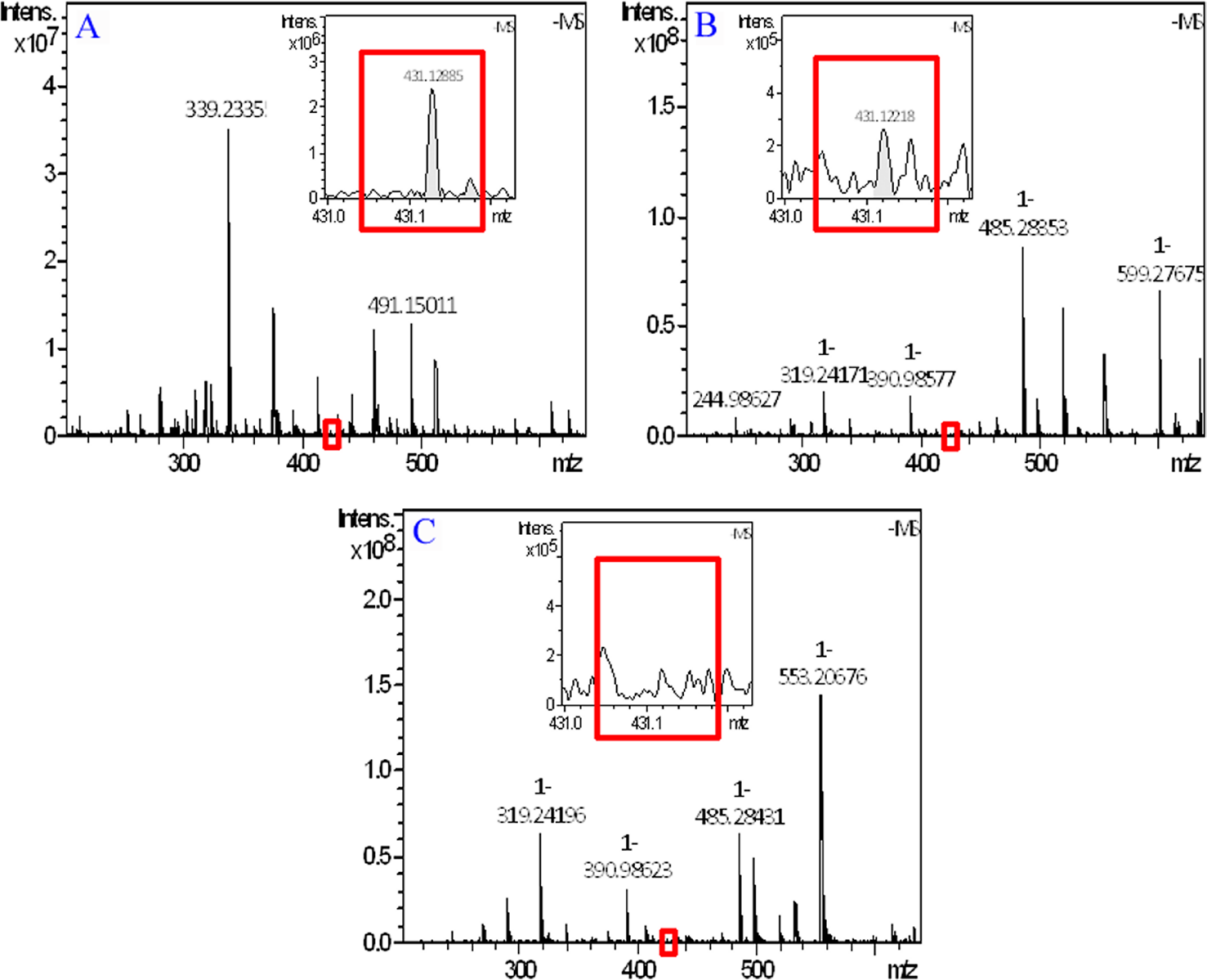
FT-MS spectrum of methanol extract of AA activated platelets with 0.1 nM THPDTPI (**A**) FT-MS spectrum of the 0.1 nM solution of THPDTPI in methanol; (**B**) FT-MS spectrum of methanol extract of AA activated platelets with 0.1 nM THPDTPI; (**C**) FT-MS spectrum of methanol extract of AA activated platelets with NS.

To evidence the adhesion of THPDTPI on the surfaces of AA activated platelets, the FT-MS spectra of 0.1 nM solution of THPDTPI in methanol (positive control), the methanol extract of AA activated platelets treated with NS (blank control), and the methanol extract of AA activated platelets treated with 0.1 nM THPDTPI were recorded and analyzed according to the literature method [19]. Figure 3A is the local amplified spectrum of 0.1 nM solution of THPDTPI in methanol. The negative ion peak at 431.12885 is from THPDTPI, and equals to its mass plus Cl. Figure 3B is the local amplified spectrum of methanol extract of AA activated platelets treated with 0.1 nM THPDTPI. The negative ion peak at 431.12218 is also from THPDTPI, and equals to its mass plus Cl. Figure 3C is the local amplified spectrum of methanol extract of AA activated platelets treated with NS. It gives no comparable negative ion peak.

### Flow cytometry supports THPDTPI inhibiting the expression of P-selectin

**Figure 4:**
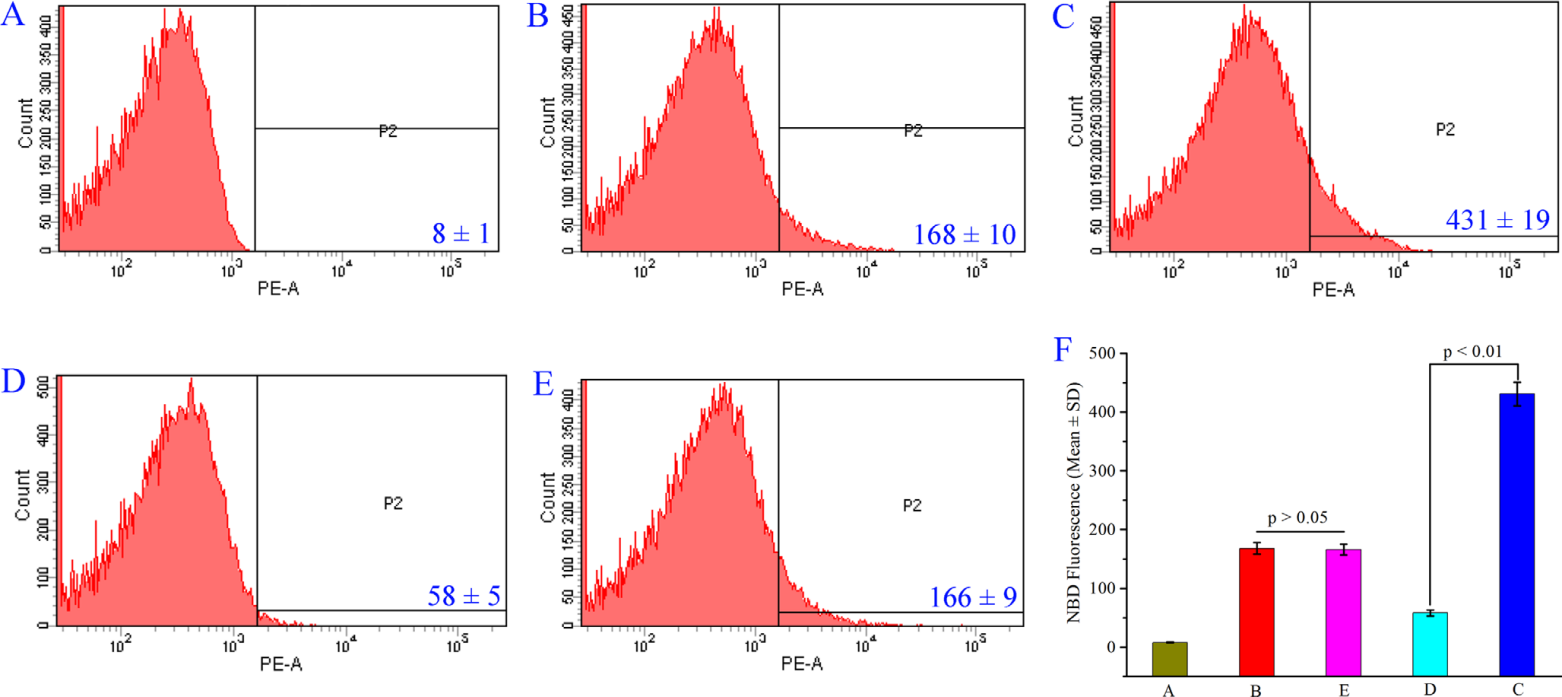
THPDTPI (0.1 nM) inhibits AA activated platelets to express P-selectin (**A**) NBD fluorescence intensity of PE-anti-CD62P unlabeled rat platelets; (**B**) NBD fluorescence intensity of PE-anti-CD62P labeled rat platelets; (**C**) NBD fluorescence intensity of PE-anti-CD62P labeled platelets activated by AA; (**D**) NBD fluorescence intensity of PE-anti-CD62P labeled platelets activated by AA with 0.1 nM THPDTPI; (**E**) NBD fluorescence intensity of PE-anti-CD62P labeled rat platelets with 0.1 nM THPDTPI; (**F**) Quantitative comparison of NBD fluorescence intensity of rat platelets in A–E, *n* = 5.

To support THPDTPI inhibiting P-selectin expression the intensity of nitrobenzoxadiazole (NBD) fluorescence was measured with flow cytometry experiments [20]. The NBD fluorescence intensity of PE-anti-CD62P unlabeled platelets (background), PE-anti-CD62P labeled platelets (reference), PE-anti-CD62P labeled and AA activated platelets without THPDTPI (positive control), PE-anti-CD62P labeled and AA activated platelets with THPDTPI (0.1 nM) and PE-anti-CD62P labeled resting platelets with THPDTPI (0.1 nM) are shown in Figure 4A–4E, respectively. The NBD fluorescence intensity of PE-anti-CD62P labeled and AA activated platelets with THPDTPI is significantly lower than that of PE-anti-CD62P labeled and AA activated platelets without THPDTPI. This means that 0.1 nM THPDTPI can effectively inhibit AA activated platelets to express P-selectin. On the other hand, the NBD fluorescence intensity of PE-anti-CD62P labeled resting platelets with THPDTPI (0.1 nM) is equal to that of PE-anti-CD62P labeled resting platelets without THPDTPI. This means that THPDTPI does not change the NBD fluorescence intensity of resting platelets. Figure 4F explains the NBD fluorescence intensities in a quantitative manner.

### UV spectra support THPDTPI inhibiting the expression of P-selectin

**Figure 5:**
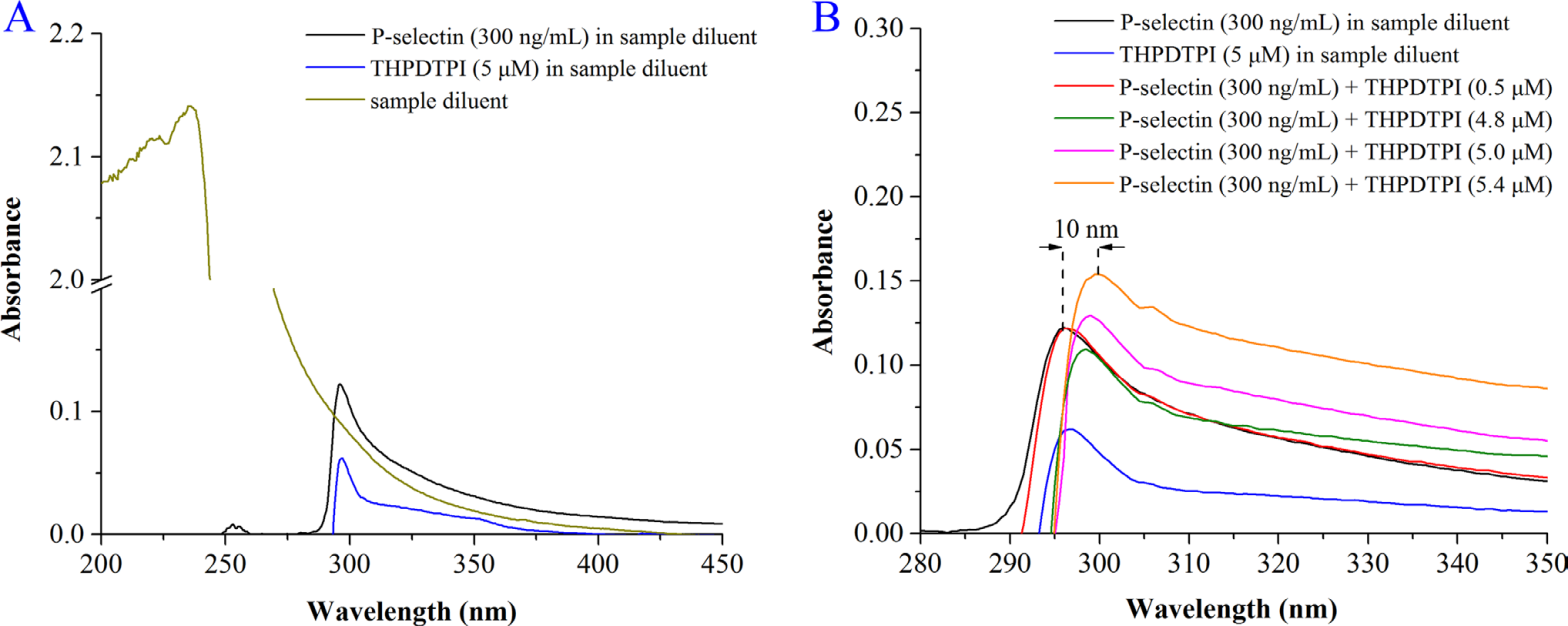
The effect of THPDTPI on the UV spectrum of P-selectin (**A**) UV spectra of the sample diluent, P-selectin in the sample diluent (300 ng/mL) and THPDTPI (5.0 μM) in the sample diluent; (**B**) UV spectra of P-selectin (300 ng/mL) and THPDTPI (final concentration 0.5, 4.8, 5.0 and 5.4 μM) in the sample diluent.

To support THPDTPI inhibiting P-selectin expression the effect of THPDTPI on the UV spectrum of P-selectin was analyzed. Figure 5A shows the UV spectra of the sample diluent, P-selectin in the sample diluent (final concentration 300 ng/mL) and THPDTPI in the sample diluent (final concentration 5.0 μM). Figure 5B shows the UV spectra of P-selectin (300 ng/mL) plus THPDTPI (0.5, 4.8, 5.0 and 5.4 μM) in the sample diluent. As seen, THPDTPI concentration-dependently induces the UV spectrum of P-selectin to have a hyperchromic effect and red shift. This suggests that THPDTPI can bind to P-selectin, thereby inhibits the expression of P-selectin.

### THPDTPI inhibits rat platelet aggregation *ex vivo*

**Figure 6:**
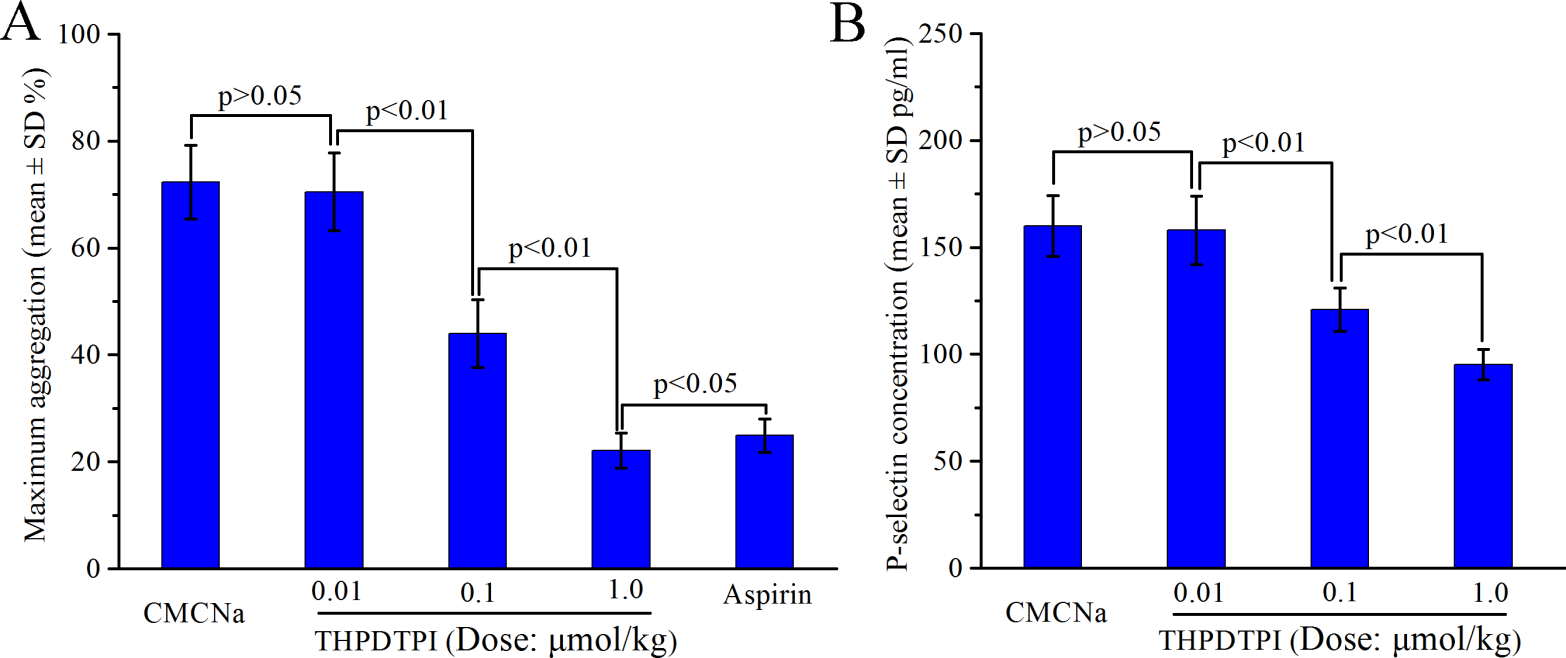
Effect of THPDTPI on platelet aggregation and sP-selectin expression of the rats receiving anti-thrombotic assay, *n* = 12 (**A**) Effect of THPDTPI on the maximum aggregation of the platelets *ex vivo*; (**B**) Effect of THPDTPI on the serum concentration of sP-selectin *in vivo*.

To show the *ex vivo* effect of THPDTPI on the platelet aggregation of the platelets of rats receiving the anti-thrombotic assay, the platelets of the rats orally treated with CMCNa (3 mL/kg), aspirin (167 µmol/kg) and THPDTPI (1.0, 0.1 and 0.01 µmol/kg) were isolated and treated with ADP (5 × 10^−6^ M) [21]. The maximum aggregations were calculated. Figure 6A indicates that THPDTPI dose dependently inhibits rat platelet aggregation. The minimal effective dose is 0.1 µmol/kg. The maximum aggregation of the platelets of the rats treated with 1.0 µmol/kg of THPDTPI (22.1 ± 3.3%) is statistically different from that of the platelets of the rats treated with 167 µmol/kg of aspirin (24.9 ± 3.1%).

### ELISA supports THPDTPI inhibiting sP-selectin expression *in vivo*

To explore the effect of THPDTPI on sP-selectin expression *in vivo*, the serum of rats receiving the anti-thrombotic assay were isolated and the concentrations of sP-selectin in the serum of the rats orally treated with CMCNa (3 mL/kg) and THPDTPI (1.0, 0.1 and 0.01 µmol/kg) were measured by using ELISA experiment. Figure 6B indicates that *in vivo* THPDTPI dose dependently inhibits sP-selectin expression. The minimal effective dose is 0.1 µmol/kg. The relative inhibition rates of 1.0, 0.1and 0.01 µmol/kg of THPDTPI to CMCNa in inhibiting sP-selectin expression are 40.5%, 24.4% and 0.01%, respectively.

### THPDTPI possesses triple activity *in vivo*

**Figure 7:**
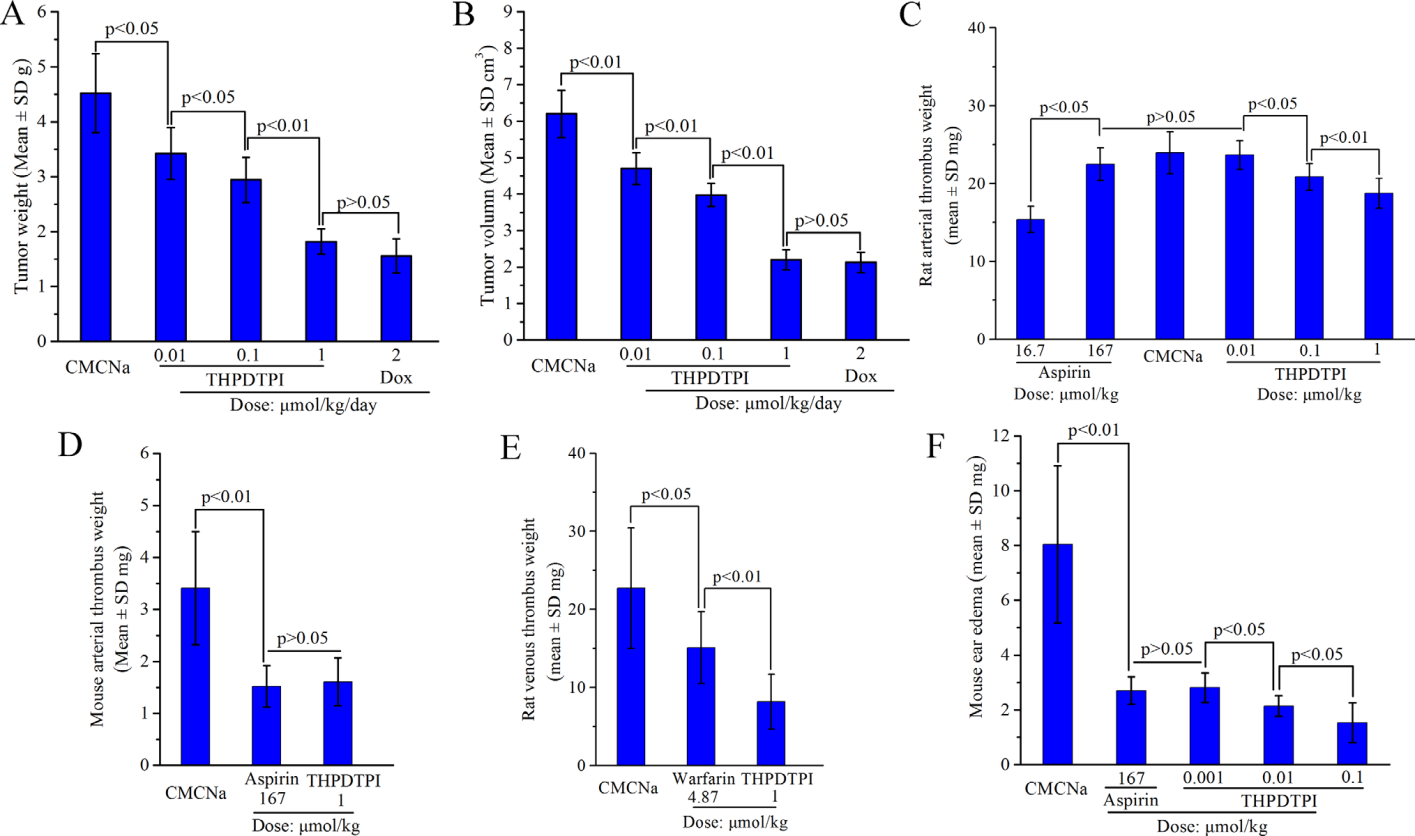
THPDTPI exhibits triple activity *in vivo*, *n* = 12 (**A**) Tumor weight of THPDTPI treated S180 mice; (**B**) Tumor volume of THPDTPI treated S180 mice; (**C**) Arterial thrombus weight of THPDTPI treated rats; (**D**) Arterial thrombus weight of THPDTPI treated mice; (**E**) Venous thrombus weight of THPDTPI treated rats; (**F**) Xylene induced ear edema of THPDTPI treated mice.

To show the benefit of targeting P-selectin, the anti-tumor, anti-thrombotic and anti-inflammatory activities of THPDTPI were evaluated *in vivo*. The anti-tumor activity of THPDTPI was evaluated on S180 mouse model according to the literature method [22]. Figure 7A and 7B indicate that the 7-day-treatment of THPDTPI leads to the slowing of tumor growth and the decreasing of tumor volume in a dose-dependent manner. The minimal effective dose is 0.01 μmol/kg/day. The anti-thrombotic activity of THPDTPI was assayed on arteriovenous shunt thrombosis model of rats according to the literature method [19]. Figure 7C indicates that the arterial thrombus weight of the rats orally receiving THPDTPI (0.01, 0.1 and 1 µmol/kg) progressively decreased with the increase of the dose. The arterial thrombus weight of the rats orally receiving 0.1 µmol/kg of THPDTPI is significantly lower than that of the rats orally receiving CMCNa. The minimal effective dose is 0.1 µmol/kg. The anti-thrombotic activity of THPDTPI was also assayed on mouse model by soaking the isolated aorta with FeCl_3_ solution according to the literature method [20]. Figure 7D indicates that the arterial thrombus weight of the mice orally receiving THPDTPI (dose, 1 µmol/kg) is equivalent to that of the mice orally receiving aspirin (dose, 167 µmol/kg). This means that the activity of THPDTPI inhibiting arterial thrombosis of mice is 167 fold higher than that of aspirin. The anti-thrombotic activity of THPDTPI was further assayed on venous thrombosis model of rats according to the literature method [23]. Figure 7E indicates that the venous thrombus weight of the rats orally receiving THPDTPI (dose, 1 µmol/kg) is significantly lower than that of the rats orally receiving warfarin (dose, 4.87 µmol/kg). This means that the activity of THPDTPI inhibiting venous thrombosis is 4.87 fold higher than that of warfarin. The anti-inflammatory activity of THPDTPI was assayed on xylene induced ear edema mouse model. Figure 7F indicates that the ear edema of the mice is dose dependently decreased by the oral THPDTPI (0.01, 0.1 and 1 µmol/kg). Besides, the ear edema of the mice orally receiving 0.001 µmol/kg of THPDTPI is significantly lower than that of the mice orally receiving CMCNa, while 0.0001 µmol/kg of THPDTPI has no effect on the ear edema. These mean that the minimal effective dose of THPDTPI inhibiting inflammation is 0.001 µmol/kg. Figure 7F also indicates that the ear edema of the mice orally receiving 0.001 µmol/kg THPDTPI is equivalent to that of the mice orally receiving 167 µmol/kg aspirin. This suggests that the activity of THPDTPI is 167000 fold higher than that of aspirin. It is worthy of mention that at 16.7 µmol/kg of dose aspirin has no effect on the ear edema, similarly at 0.0001 µmol/kg of dose THPDTPI has no effect on the ear edema. Therefore the *in vivo* assays demonstrate that on 5 models THPDTPI possesses triple activity, i.e. the anti-tumor activity, the anti-thrombotic activity and the anti-inflammatory activity. This can generally attribute to THPDTPI adhering to platelet, acting on the active site of P-selectin and inhibiting the expression of P-selectin.

### THPDTPI downregulates serum IL-8 and TNF-α *in vivo*

**Figure 8:**
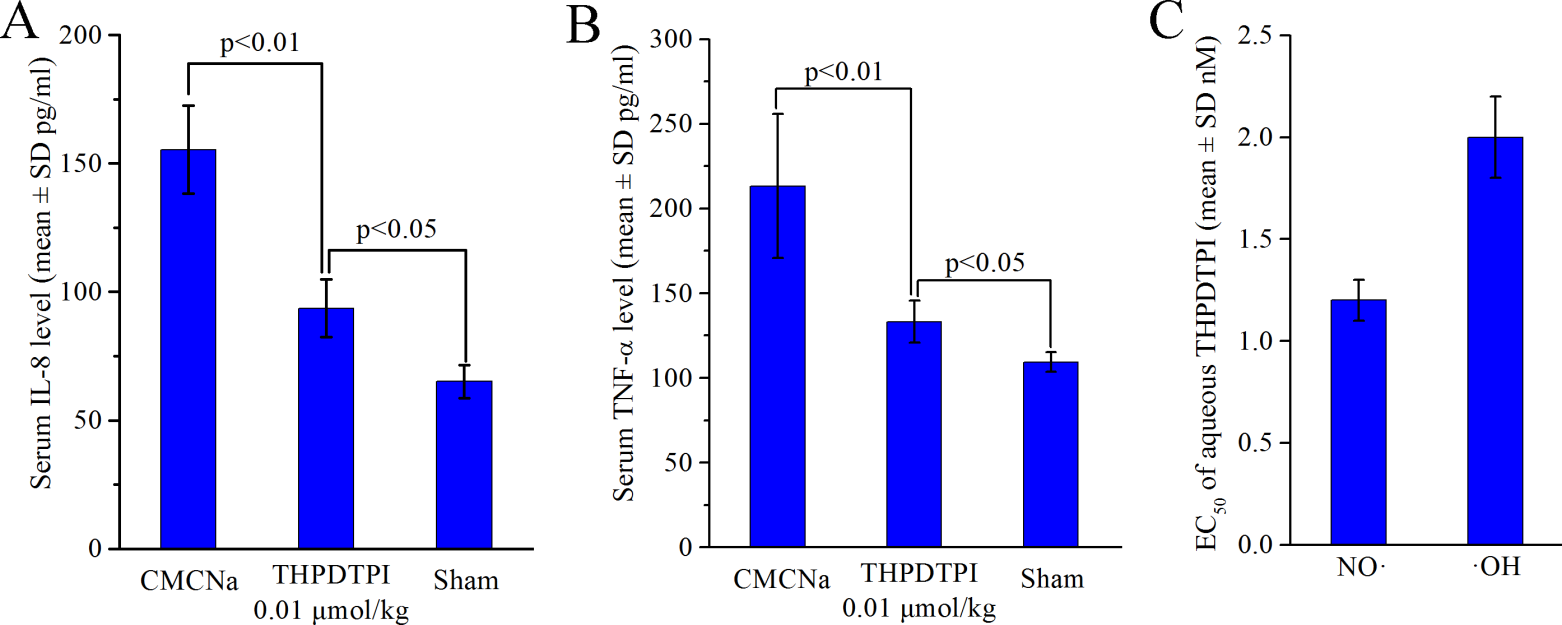
THPDTPI downregulating serum IL-8 and TNF-α *in vivo*, as well as scavenging NO· free radicals and ·OH free radicals *in vitro* (**A**) *In vivo* THPDTPI downregulating serum IL-8 at a dose of 0.01 µmol/kg, *n* = 12; (**B**) *In vivo* THPDTPI downregulating serum TNF-α at a dose of 0.01 µmol/kg, *n* = 12; (**C**) EC_50_ values of the aqueous THPDTPI scavenging NO· free radicals and ·OH free radicals *in vitro*; *n* = 5.

In inflammatory diseases the upregulation of sP-selectin, TNF-α and IL-8 has been widely noticed [24, 25]. To show the benefits of THPDTPI adhering on platelets and targeting P-selectin, the concentration of IL-8 and TNF-α in the serum of the ear edema mice were measured [26]. Figure 8A and 8B indicate that the serum IL-8 and TNF-α of ear edema mice orally receiving THPDTPI (dose, 0.001 µmol/kg) are significantly lower than those of ear edema mice orally receiving CMCNa. This means that at the dose of 0.001 µmol/kg THPDTPI can downregulate the serum IL-8 and TNF-α *in vivo*.

### THPDTPI scavenges NO· and ·OH free radicals

The thrombosis, tumor growth, inflammation and P-selectin expression were well correlated through NO· free radicals and ·OH free radicals [27–29]. For this profile the efficacy of THPDTPI scavenging NO· free radicals and ·OH free radicals were evaluated. Figure 8C indicates that the EC_50_ of aqueous THPDTPI scavenging NO· free radicals and ·OH free radicals are 1.2 ± 0.1 nM and 2.0 ± 0.2 nM, respectively.

## DISCUSSION

The relevant high level of sP-selectin enhances the tumor growth [30], worsens the thrombotic diseases [31], and promotes the attack of inflammatory diseases [32]. The discovery of the downregulator and/or inhibitor of P-selectin are of clinical importance. The docking of 4 classes of anti-tumor β-carbolines and 3 classes of anti-thrombotic tetrahydro-β-carbolines from our sample library towards the active site of P-selectin led to the rational design, and THPDTPI was theoretically assigned a promising inhibitor of P-selectin. Recently the importance of the interactions between the inhibitor with Tyr48 and Tyr94 residues of the active site of P-selectin were disclosed [33]. The docking showed that THPDTPI can target P-selectin by forming π-π interactions with Tyr44, Tyr48 and Tyr94 residues of its active site, and in turn the emphasis was placed on the contribution of these π-π interactions to THPDTPI targeting P-selectin. This theoretical issue was supported by a series of experiments and evaluation. In UV experiment THPDTPI induced the spectrum of P-selectin having a hyperchromic effect and red shift. In flow cytometry experiment THPDTPI decreased the NBD fluorescence intensity of PE-anti-CD62P labeled and AA activated platelets. In the *in vivo* evaluation the concentration of sP-selectin in the serum of the rats receiving anti-thrombotic assay was dose dependently lowered by THPDTPI. Thus the theoretical profile, the UV experiment, the flow cytometry experiment and the anti-thrombotic evaluation together showed that by the targeting action THPDTPI inhibited the expression of P-selectin.

To target and thereby to inhibit P-selectin expression THPDTPI needs to adhere on the surface of the platelets. This was characterized by AFM and FT-MS experiments. Even though the AFM images visualized that adhering onto the surface of the platelets been an inherent property of the nanoparticles of THPDTPI, only the nanoparticles on the surface of AA activated platelets, but not the resting platelets, can form the stable adhesion. This stable adhesion was supported by FT-MS experiment. The FT-MS spectrum of methanol extract of AA activated platelets incubated with THPDTPI, but not the resting platelets incubated with THPDTPI, gave a molecular ion peak of THPDTPI. The AFM images also visualized that only the stable adhesion resulted in the AA activated platelets to lose the aggregation potency and to decrease the number of extended pseudopodia. AFM and FT-MS together suggest that the adhesion of THPDTPI on the surfaces of AA activated platelets is essential for targeting and thereby for inhibiting P-selectin expression.

The benefits of THPDTPI targeting and thereby inhibiting P-selectin expression can be mirrored in the biological events, and were characterized by anti-tumor, anti-thrombotic and anti-inflammatory actions, the so called triple action here. On S180 mouse model THPDTPI was ensured to be able to effectively slow the tumor growth. The minimal effective dose of THPDTPI inhibiting tumor growth was 0.01 μmol/kg. On rat arteriovenous shunt thrombosis model and on mouse model of FeCl_3_ solution soaking aorta, THPDTPI was ensured to be able to effectively inhibit the formation of the arterial thrombus. The minimal effective dose of THPDTPI inhibiting the arterial thrombosis was 1 μmol/kg. On rat left inferior vein ligatured model THPDTPI was ensured to be able to effectively inhibit the formation of the venous thrombus. The minimal effective dose of THPDTPI inhibiting the venous thrombosis was 1 μmol/kg. On xylene induced mouse ear edema model THPDTPI was ensured to be able to effectively inhibit ear edema. The minimal effective dose of THPDTPI inhibiting the inflammation was 0.001 μmol/kg. These data suggest that THPDTPI targets and inhibits P-selectin expression may lead to the triple action, i.e. the anti-tumor, the anti-thrombotic and the anti-inflammatory actions. The common effective dose of the triple action is 1 μmol/kg.

The triple action also can correlate with THPDTPI been able to scavenge the free radicals and lower the levels of IL-8 and TNF-α in mouse serum [29, 34, 35]. *In vitro* the EC_50_ values of THPDTPI scavenging NO· free radicals and ·OH free radicals were 1.2 nM and 2.0 nM, respectively. *In vivo* the serum levels of IL-8 and TNF-α of the ear edema mice could be significantly lowered by 0.001 μmol/kg of THPDTPI. Consequently, the triple action can attribute to the inhibition of cell adhesion induced by THPDTPI targeting P-selectin.

## MATERIALS AND METHODS

### General

Male Sprague Dawley rats (250–300 g) and male ICR mice (20–22 g) were purchased from the Laboratory Animal Center of Capital Medical University. All evaluations were based on the protocol reviewed and approved by Ethics Committee of Capital Medical University. The committee assured that the animal welfare was maintained in accordance to the requirements of Animal Welfare Act and NIH Guide for Care and Use of Laboratory Animals. Statistical analyses of all biological data were carried out by use of ANOVA, and LSD for multiple group comparison. All analyses were done with SPSS 19.0 program, and *p*-value < 0.05 was considered statistically significant. THPDTPI was self-prepared by following our procedure [8].

### Generation of 3D conformation of THPDTPI

The 2D structure of THPDTPI was sketched in ChemDraw Ultra 10.0, converted to 3D conformation in Chem3D 10.0, and then energy minimized in Discovery Studio 3.5 with a Merck molecular force field (Merck & Co.) until the minimum RMS reached 0.001. The energy optimized conformations in the whole conformational space of THPDTPI were sampled with systematic search and BEST method of Discovery Studio 3.5, which were practiced with a SMART minimizer using CHARMM force field. The energy threshold was set to 20 kcal/mol at 300 K. The maximum minimization steps were 200 and the minimization root mean squared (RMS) gradient was 0.1 Å. The maximum generated conformations were 255 with a RMS deviation (RMSD) cutoff of 0.2 Å. Top 10 energy optimized conformations of THPDTPI were used for the docking to the active site of P-selectin.

### Docking investigation

Software AutoDock 4 was used to perform the molecular docking of 10 energy optimized conformations of THPDTPI toward the active site of P-selectin. The average structure of P-selectin was started from PDB entry 1G1R and obtained via molecular dynamics simulation. P-Selectin was treated as rigid and prepared by AutoDockTools 1.5, i.e. merging nonpolar hydrogen, assigning gasteiger charge and autodock element. Ten energy optimized conformations of THPDTPI were treated as rigid ligand and prepared by AutoDockTools 1.5, i.e. merging nonpolar hydrogen, assigning gasteiger charge, finding root and aromatic carbon, detecting rotatable bond, and setting torsion. The grid box dimensions were set to 50 Å × 50 Å × 50 Å with a grid spacing of 0.375 Å. The grid box center was set to the Ca^2+^ in the crystal structure PDB 1G1R. Lamarckian Genetic Algorithm (LGA) was used to find the appropriate binding position, orientation and the conformation of THPDTPI in the active site of P-selectin. The global optimization was started with parameters of a population of 300 randomly positioned individuals. The maximum number of energy evaluation was increased to 2.5 × 10^7^, and the maximum number of generations in LGA was increased to 2.7 × 10^5^. The Solis and Wets local search was performed with a maximum number of 3000. During molecular docking 200 runs were carried out for THPDTPI. The resulted 200 conformations of THPDTPI were scored by the lowest binding energy and clustered with an rms tolerance of 2.0 Å.

### Flow cytometry based P-selectin expression assay

Sprague Dawley rat blood was collected in an aqueous solution of 3.8% sodium citrate (1:9, v/v) and immediately centrifuged at 160 g for 15 min to collect platelet-rich-plasma (PRP). To 500 µL of PRP, 10 µL of THPDTPI (final concentration, 0.1 nM) was added and the mixture was incubated at 37°C for 30 min, to which 10 µL of AA (Sigma-Aldrich Corp, St Louis MO, USA) in NS (0.15 mg/mL) was added. The mixture was incubated for 5 min at 37°C and centrifuged at 500 g for 10 min to allow the platelets to precipitate. The platelets were stained with phycoerythrin (PE)-anti-CD62P (Shanghai XingYou Biological Technology Co., Ltd., Shanghai, P. R. China) for 20 min. PE fluorescence for gated platelet populations was analyzed for 10,000 events (counts) per sample aliquot, and fluorescence data were recorded accordingly. The level of platelet activation was assessed by the fluorescence height of P-selectin.

### UV spectra defined interaction between THPDTPI and P-selectin

The sample diluents for P-selectin UV spectrum were from rat ELISA kit (Rat P-selectin ELISA Kit; Wuhan Huamei Biotech Co., Ltd., Wuhan, Hubei Province, P. R. China). A solution of P-selectin in sample diluents was prepared (300 ng/mL), from which 300 μL was added into six eppendorf tubes. The control tube only contains 300 μL of sample diluents. The sample tube contains 300 μL solution of P-selectin in sample diluents (300 ng/mL) and 10 μL solution of THPDTPI in sample diluents (final concentration: 0.5, 4.8, 5.0 and 5.4 μM). All tubes were incubated at room temperature for 12 h then received UV test on a Shimadzu 2550 spectrophotometer.

### FT-MS defined interaction between THPDTPI and platelets

To explore the action of THPDTPI on platelet electrospray ionization(-)/fourier transform mass spectrometry (ESI(-)/FT-MS) spectra were recorded for monitoring the accumulation of THPDTPI on AA activated platelets. The PRP were collected by following the procedure in flow cytometry assay. To 500 µL PRP 10 µL solution of THPDTPI in NS (final concentration, 0.1 nM) or NS was added and the mixture was incubated at 37°C for 30 min, to which 10 µL solution of AA in NS (0.15 mg/mL) was added. The mixture was incubated at 37°C for 5 min and centrifuged at 500 g for 10 min to precipitate the platelets, after the removal of the supernatant the platelets were washed with NS for 3 times, in 200 µL methanol the residue received 10-min ultrasonic disintegration, the mixture was at 500 g centrifuged for 10 min to separate the supernatant for FT-MS analysis.

### AFM image defined interaction between THPDTPI and platelets

Rat blood and 3.8% aqueous sodium citrate (9/1, v/v) was centrifuged at 160 g for 10 min to collect the PRP. The PRP was centrifuged for 10 min at 300 g to collect the precipitates. To the precipitates 1.5 mL NS was added, the mixture was centrifuged (300 g, 10 min) to get resting platelets (1 × 10^5^/mL). The resting platelets were incubated with THPDTPI (final concentration, 5 × 10^−8^, 5 × 10^−9^ and 5 × 10^−10^ M) or NS at 37°C for 30 min. The resting platelets were activated with AA (final concentration 350 mM) at 37°C for 5 min. AA activated platelets were incubated with THPDTPI (final concentration, 5 × 10^−8^, 5 × 10^−9^ and 5 × 10^−10^ M) or NS at 37°C for 30 min. The platelet samples were individually dropped onto a mica sheet, fixed with glutaraldehyde (3%) for 10 min, carefully washed with ultrapure water, and dried in air. With the contact mode atomic force microscopy (AFM) images of all platelet samples and THPDTPI in NS (5 × 10^−8^, 5 × 10^−9^ and 5 × 10^−10^ M) were recorded on a Nanoscope 3D AFM (Veeco Metrology, Santa Barbara, CA, USA) in ambient conditions.

### *In vivo* anti-tumor assay

S180 cells (purchased from Vital River Laboratory Animal Technology Co., Ltd. Beijing, China) were xenografted subcutaneously in male ICR mice (20–22 g). The mice were housed in sterile isolated cages at constant temperatures (22–25°C) and allowed to take food and water freely. Tumor growth was monitored by measuring the volume with a caliper, which was calculated based on Volume (cm^3^) = Length × Width^2^/2. When the average volume of tumors xenograft reached 0.5 cm^3^ the mice were randomly divided into treatment groups (12 per group). The mice were orally treated with THPDTPI (0.01, 0.1 and 1.0 μmol/kg/day) for 7 consecutive days, or intraperitoneally treated with doxorubicin (Dox, positive control, 2 μmol/kg/day) for 7 consecutive days, or orally treated with 0.5% carmellose sodium (CMCNa, vehicle, negative control, 0.2 mL/mouse/day) for 7 consecutive days. Mice were weighed daily. Twenty-four hours after the last administration, mice were weighed, sacrificed by ether anesthesia, and dissected to immediately obtain the tumors for testing their volume and weight.

### Rat arterial thrombus weight assay-arteriovenous shunt model

Male SD rats (250 to 300 g) were randomly divided into groups (each 12).Thirty minutes after orally giving 0.5% CMCNa (3 mL/kg) or suspension of aspirin in 0.5% CMCNa (dose, 16.7 and 167 µmol/kg) or suspension of THPDTPI in 0.5% CMCNa (dose, 1, 0.1 and 0.01 µmol/kg) the rats intraperitoneally received pentobarbital sodium anesthesia (80.0 mg/kg) to separate the left jugular vein and the right carotid artery. Polyethylene tubes were used to make shunts between the jugular vein and carotid artery. Into the middle polyethylene tube a weighed 6-cm non-absorbable surgical thread of silk was put, and a solution of heparin sodium in NS (50 IU/mL) was injected as anticoagulant to fill the tube. The ends of the tube were inserted into the left jugular vein and the right carotid artery, and via the tube the blood flowed from the right carotid artery to the left jugular vein for 15 min. The surgical thread was taken out to weigh the thrombus weight. The blood was sampled for platelet aggregation assay *ex vivo* and ELISA based *in vivo* P-selectin expression assay.

### Platelet aggregation assay *ex vivo*

The blood from rat arterial thrombus weight assay was drawn into a syringe with 3.8% sodium citrate. The PRP was prepared by centrifuging the blood at 160 g for 10 min, and remaining blood was centrifuged for an additional 10 min at 240 g to prepare the PPP. The count of the platelets in the PRP was adjusted to 2 × 10^8^ platelets/mL with autologous PPP. Into the optical aggregometry testing tuber 0.5 μL of adjusted PRP of the rats receiving 0.5% CMCNa (3 mL/kg) or suspension of aspirin in 0.5% CMCNa (dose, 16.7 and 167 µmol/kg) or suspension of THPDTPI in 0.5% CMCNa (dose, 1, 0.1 and 0.01 µmol/kg) was added. The baseline was adjusted, 5μL solution of ADP in NS (final concentration 10 μM) was added, and the aggregation was tested at 37°C for 5 min. The tests in six plicate tubers were performed. The maximum platelet aggregation was represented by peak height of aggregation curve.

### ELISA based *in vivo s*P-selectin expression assay

The assay was performed by using ELISA kit (Rat P-selectin ELISA kit, Cusabio Biotech Co., USA) and the 96-well plate coated with the enzyme. The blood from rat arterial thrombus weight assay was sampled to prepare rat serum. The well containing 980 μL of the serum of rats orally treated with 0.5% CMCNa (3 mL/kg) or 980 μL of the serum of rats orally treated with the suspension of THPDTPI in 0.5% CMCNa (dose, 1.0, 0.1 and 0.01 µmol/kg) were incubated at 37°C for 3 min. The plate was incubated at 37°C for 120 min, and the solvent was removed. To each well 100 μL of biotin labeling antibody (from the kit) were added, incubated at 37°C for 60 min, the solvent was removed, and washed for three times. To each well 100 μL of horseradish peroxidase labeling avidin were added, incubated at 37°C for 60 min, the solvent was removed and washed five times. To each well 90 μL substrate was added, incubated at 37°C in dark for 20 min-coloration. To each well 50 μL of stop solution was added to stop the coloration. Within 15 min the OD values of the wells were tested at 450 nm and the concentration of P-selectin from the platelets of CMCNa and THPDTPI orally treated rats were calculated.

### Arterial thrombus weight assay-mouse model

Male ICR mice (20–22 g) were randomly divided into groups (each 12). Thirty minutes after orally giving 0.5% CMCNa (10 mL/kg) or suspension of aspirin in 0.5% CMCNa (dose, 167 µmol/kg) or suspension of THPDTPI in 0.5% CMCNa (dose, 1 µmol/kg) the mice were anesthetized with chloral hydrate (10 g/100 mL, intraperitoneally) and 1 cm of segment abdominal aorta was dissected. Then, thrombosis was triggered immediately by way of vessel damage, i.e. the artery was wrapped in gauze strips (0.5 cm in width and 3 cm in length) saturated with 25% ferric chloride solution for 15 min, 0.5 cm of artery segments with thrombi formation were excised, and the weights of the thrombus were determined.

### Venous thrombus weight assay-rat model

Male SD rats (250 to 300 g) were randomly divided into groups (each 12) and fasted for 24 h before dosing. Thirty minutes after orally giving 0.5% CMCNa (3 mL/kg)or a suspension of warfarin in 0.5% CMCNa (dose, 4.87 µmol/kg) or a suspension of THPDTPI in 0.5% CMCNa (dose, 1 µmol/kg) the rats successively received intraperitoneal pentobarbital sodium anesthesia (80.0 mg/kg) and midline laparotomy. Briefly, through a midline cervical incision, the vein of left inferior vena cava just below the renal veins was identified, dissected, isolated from its surrounding structures and ligatured with silk suture. At this point the skin was closed and the rats were allowed to recover for 4 h. The rats were reanesthetized, the incision was reopened and the vein was once again isolated and then was excised. The thrombus was meticulously extracted from the vein and weighed.

### Anti-inflammation assay

Male ICR mice (20–22 g) were randomly divided into 7 groups (each 12). The mice were orally given a single dose of 0.5% CMCNa (10 mL/kg), or a suspension of aspirin in 0.5% CMCNa (dose, 167 and 16.7 μmol/kg) or the suspensions of THPDTPI in 0.5% CMCNa (dose, 0.1, 0.01, 0.001 and 0.0001 µmol/kg). Thirty minutes later, 0.03 mL of xylene was applied to both the anterior and posterior surfaces of the right ear. The left ear was used as control. Two hours after xylene application the mice received ether anesthesia and were sacrificed to remove both ears. By using a cork borer of 7 mm in diameter the circular sections were taken and weighed. Xylene induced increase was reflected by subtracting the weight of the untreated left ear section from that of the treated right ear section. The blood of the mice treated with 0.5% CMCNa and the suspension of THPDTPI in 0.5% CMCNa (dose, 0.01 µmol/kg) was sampled for serum TNF-α and IL-8 assay.

### Serum TNF-*α* and IL-8 assay

To prepare the serum sample 1 mL blood of healthy mice or inflammatory mice treated with CMCNa or THPDTPI (0.01 µmol/kg) was drawn into a syringe with 100 μL aqueous sodium citrate (3.8%). Blood was centrifuged at 4°C (3000 g) for 30 min to prepare serum sample. For serum TNF-α assay, to each of 3 blank wells nothing was added, to each of 6 standard wells 50 μL standard solution and 100 μL HRP-conjugate was added, to each 3 testing wells 10 μL serum of healthy mouse or inflammatory mouse treated with CMCNa or THPDTPI (0.01 µmol/kg) 40 μL sample diluents and 100 μL HRP-conjugate reagent were successively added. The well was aspirated, washed with 400 μL wash solution via a squirt bottle and the liquid was completely moved, which was repeated for 5 times. For serum IL-8 assay, to each blank well 100 μL sample diluent was added, to each standard well 50 μL standard solution and 50 μL streptavidin-HRP were added, to each testing well 40 μL serum of healthy mouse or inflammatory mouse treated with CMCNa or inflammatory mouse treated with THPDTPI (0.01 µmol/kg) 10 μL anti-IL-8-antibody and 50 μL streptavidin-HRP were successively added. The plate was inverted, blotted with clean paper towels, closed with plate membrane and incubated at 37°C for 60 min. Upon removal of the membrane the liquid was discarded and the well was dried by swing. To the residue sufficient washing buffer, a buffer of the wash solution diluted with 30-fold water, was added, stilled for 30 s and then drained (this procedure was repeated for 5 times), the well was dried by pat, to the residue 50 μL chromogen solution A and B were added and gently mixed and at 37°C in the dark incubated for 10 min. To the well 50 μL stop solution was added, and the color in the well changed from blue to yellow. The plate was read at 450 nm using a microtiter plate reader within 15 min to record OD value. By using the standard curves the serum concentrations of TNF-α and IL-8 were calculated.

### NO∙ free radical scavenging assay

On JES300 Electron Spin Resonance (JES300ESR, Japan Electron Optics Laboratory Co., LDT) the signal of NO· free radical produced by the reaction of 5 µL solution of 7.325 mg N-methyl-D-glucamine dithiocarbamate (MGD; Sigma-Aldrich Corp, St Louis MO, USA) in 1 mL ultrapure water (25 mM), 5 µL solution of 3.475 g FeSO_4_·7H_2_O (Sinopharm Chemical Reagent Beijing Co., Ltd., Beijing,China) in 1 mL ultrapure water (12.5 mM) and 5 µL solution of 0.25 mg S-nitroso-N-acetyl-DL-penicillamine (SNAP; Sigma-Aldrich Corp, St Louis MO, USA) in 1 mL ultrapure water (1.1 µM) was measured. The height of the signal was calculated and defined as the blank height of NO· signal (BHNO). The effect of THPDTPI on NO· signal was defined by the signal of NO· formed with 5 µL solution of 7.325 mg of MGD in 1 mL ultrapure water (25 mM), 5 µL solution of 3.475 g of FeSO_4_·7H_2_O in 1 mL ultrapure water (12.5 mM), 5 µL solution of 0.25 mg of SNAP in 1 mL of ultrapure water (1.1 µM) and 5 µL solution of THPDTPI in ultrapure water (final concentration, 1.0, 1.5, 2.0 and 2.5 nM). The height of NO· signal was calculated and defined as THPDTPI-treated height of NO· signal (THNO·). The NO· scavenging ratio was calculated according to ‘Scavenging ratio = (BHNO⋅ - THNO⋅)/BHNO·’.

### ·OH free radical scavenging assay

On JES300 Electron Spin Resonance (JES300ESR, Japan Electron Optics Laboratory Co., LDT), the signal of ·OH free radical produced by the reaction of 2.5 µL solution of 2.78 g of FeSO_4_·7H_2_O in 1 mL ultrapure water (10 mM), 2.5 µL solution of 11.32 mg of DMPO in 1 mL ultrapure water (0.1 M) and 5 µL H_2_O_2_ (Sinopharm Chemical Reagent Beijing Co., Ltd.; 100 mM) was measured. The height of ·OH signal was calculated and defined as the blank height of the ·OH signal (BH·OH). The effect of THPDTPI on ·OH signal was defined by the signal of ·OH signal formed by 2.5 µL solution of 2.78 g of FeSO_4_·7H_2_O in 1 mL ultrapure water (10 mM), 2.5 µL solution of 11.32 mg of 5,5-dimethyl-1-pyrroline-1-oxide (DMPO), a spin-trapping agent, in 1 mL ultrapure water (0.1 M), 5 µL H_2_O_2_ (100 mM) and 5 µL solution of THPDTPI in ultrapure water (final concentration, 1.0, 1.5, 2.0 and 2.5 nM). The height of ·OH signal was calculated and defined as THPDTPI-treated height of ·OH signal (TH·OH). The ·OH scavenging ratio was calculated according to ‘Scavenging ratio = (TH·OH - BH·OH)/BH·OH’.

## CONCLUSIONS

To show the clinical prospect of P-selectin inhibition, THPDTPI was rationally designed as a nano-scaled inhibitor of P-selectin. The rationality of the design was evidenced by a series of assays. All assays were properly performed to demonstrate the impact of the downregulation of P-selectin expression to the slowing of tumor growth, to the decrease of arterial and venous thrombus weight, and to the attenuation of the inflammatory response. The process of the downregulation of P-selectin expression was experimentally explored by THPDTPI adhering onto the surfaces of AA activated platelets, entering into the active site of P-selectin, forming hydrogen bonds with the amino acid residues of the active site, changing the conformation of sP-selectin, and decreasing the serum levels of sP-selectin. This process also gave us an insight into the potential of P-selectin inhibitor to simultaneously inhibit tumor growth, thrombus formation and inflammatory response.

## SUPPLEMENTARY MATERIALS FIGURES


