# Supplementary material for: Docking of THPDTPI: to explore P-selectin as a common target of anti-tumor, anti-thrombotic and anti-inflammatory agent
Source: Oncotarget. 2017 Jul 19;9(1):268–81. doi: 10.18632/oncotarget.19374 (PMC5787463; doi:10.18632/oncotarget.19374)
Supplement: Supplementary file 1 [file oncotarget-09-268-s001.pdf]

# Docking of THPDTPI: to explore P-selectin as a common target of anti-tumor, anti-thrombotic and anti-inflammatory agent

## SUPPLEMENTARY MATERIALS

### FT-MS analysis

To explore the difference between the adhesion of THPDTPI with the resting platelets and AA activated platelets a FT-MS analysis was performed. The resting platelets and AA activated platelets incubated with $5 \times 10^{-8}$ M THPDTPI were sufficiently washed with ultrapure water, centrifuged at 500 g for 10 min, received ultrasonication in methanol for 10 min, centrifuged at 500 g for 10 min and the methanol extracts were analyzed with FT-MS. It was found that the methanol extract of AA activated platelets, but not the methanol extract of the resting platelets, gave a negative ion peak at 431.13315, the mass of THPDTPI plus Cl. Therefore only the adhesion of THPDTPI with AA activated platelets is stable enough. This difference could be attributed to P-selectin is stored in α-granules of the resting platelets but occurs on the surface of AA activated platelets. FT-MS spectrograms are provided as Supplementary Figures 1 and 2.

### *In vitro* anti-platelet aggregation activity assay of THPDTPI

An H-10 cell counter was used to determine the platelet count and a two-channel Chronolog aggregometer was used to evaluate the platelet aggregation. The blood of SD rat was centrifuged at 160 g for 10 min to collect the PRP. The remaining blood was centrifuged for an additional 10 min at 240 g to prepare platelet-poor-plasma (PPP). The final count of the platelets in the PRP was adjusted to $2 \times 10^8$ platelets/mL with autologous PPP. Into the optical aggregometry testing tuber, 0.5 µL of adjusted PRP and 5 µL of NS or 5 µL of the solution of THPDTPI in NS (final concentration: $5 \times 10^{-7}$, $5 \times 10^{-8}$, $5 \times 10^{-9}$, $5 \times 10^{-10}$ and $5 \times 10^{-11}$ M) was added. The baseline was adjusted, and 5 µL solution of ADP in NS (final concentration 10 µM) was added. The aggregation was tested at 37°C for 5 min. The effect of THPDTPI on ADP-induced platelet aggregation was identified. The tests in six plicate tubers were performed. The maximum platelet aggregation (*Am*) of NS or THPDTPI was represented by peak height of aggregation curve. The inhibition rate was calculated by following Inhibition (%) = [(*Am* of NS) - (*Am* of THPDTPI)]/(*Am* of NS) × 100%. *Am*% of NS was 48.65 ± 3.12%. The concentration vs. inhibition rate curve was plotted to determine the $IC_{50}$ value with GWBASIC. EXE program. The $IC_{50}$ value of THPDTPI against ADP-induced platelet aggregation was 0.26 nM.

### *In vivo* bleeding time assay

Male ICR mice (20 ± 2 g) were randomly divided into treatment groups (12 per group). The mice were orally treated with THPDTPI (0.01, 0.1 and 1.0 µmol/kg/day) for 10 days, or orally treated with 0.5% carmellose sodium (CMCNa, vehicle, negative control, 0.2 mL/mouse/day) for 10 days. Mice were weighed daily. Twenty-four hours after the last administration, the mice received tail bleeding time assay by following a standard procedure. Briefly, the mouse was placed in a tube holder and its tail was out of the holder. A 2 mm cut was made on the tail. The flowing blood was wiped away with a filter paper every 30 s until it stopped. The yielded bleeding time of the mice treated with 1.0 µmol/kg/day, 0.1 µmol/kg/day and 0.1 µmol/kg/day of THPDTPI as well as 0.2 mL/mouse/day of 0.5% CMCNa were 1622.17 ± 274.43 sec, 1600.17 ± 223.09 sec, 1556.70 ± 226.49 sec and 1636.70 ± 250.19 sec, respectively. THPDTPI did not cause prolonged bleeding time.

### Determination of HPLC purity

An Agilent Technologies 1200 Series HPLC (high performance liquid chromate graphy) system (Agilent Technologies, Santa Clara, CA, USA) was used. The sample was separated on a Waters XTerra C18 reversed-phase column (2.1 × 150 mm, 5 µm; Waters Limited, Hertfordshire, UK) protected by a guard column of the same material (5 × 10 mm, 5 µm). The column thermostat was maintained at 40°C. To the column, 5 µL of a solution of THPDTPI in ultrapure water was injected for analysis. The mobile phase consisted of water and acetonitrile (5/95). The flow rate was 0.2 mL/min. The column was washed with water and methanol (35/65), and equilibrated to initial conditions for 15 min. Ultraviolet (UV) absorption spectra were recorded online. The UV detector was set to a scanning range of 200–400 nm, and a wavelength of 254 nm was used to monitor THPDTPI. The chromatogram was recorded and gave THPDTPI a retention time of 5.704 min and 98.9% purity.

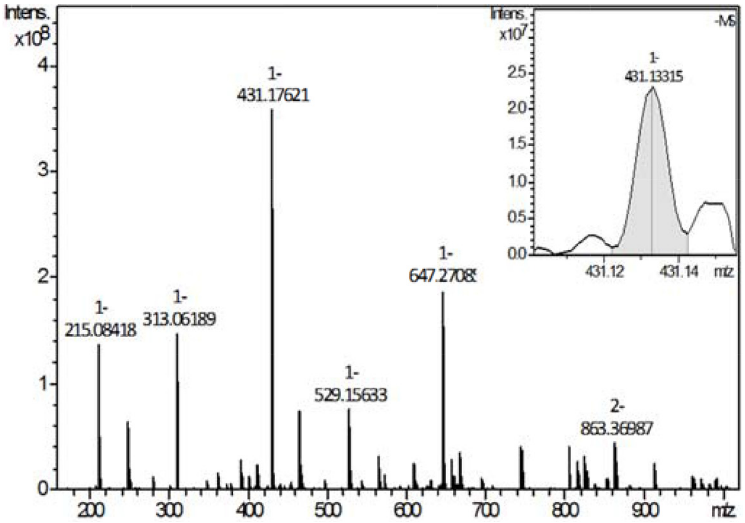


**Supplementary Figure 1: ESI(-)-FT-MS of the methanol extract of AA activated platelets with THPDTPI.** The local amplified spectrogram gives a negative ion peak at 431.13315.

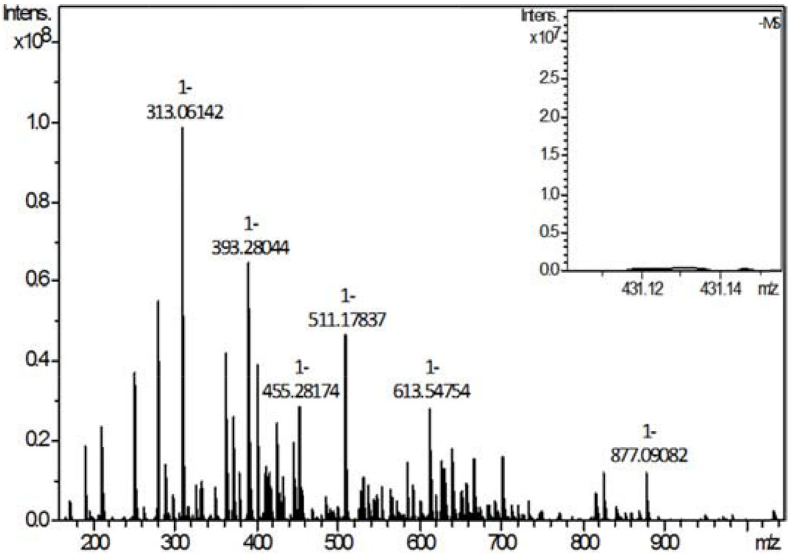


**Supplementary Figure 2: ESI(-)-FT-MS of the methanol extract of the resting platelets with THPDTPI.** The local amplified spectrogram gave no negative ion peak at 431.13315.

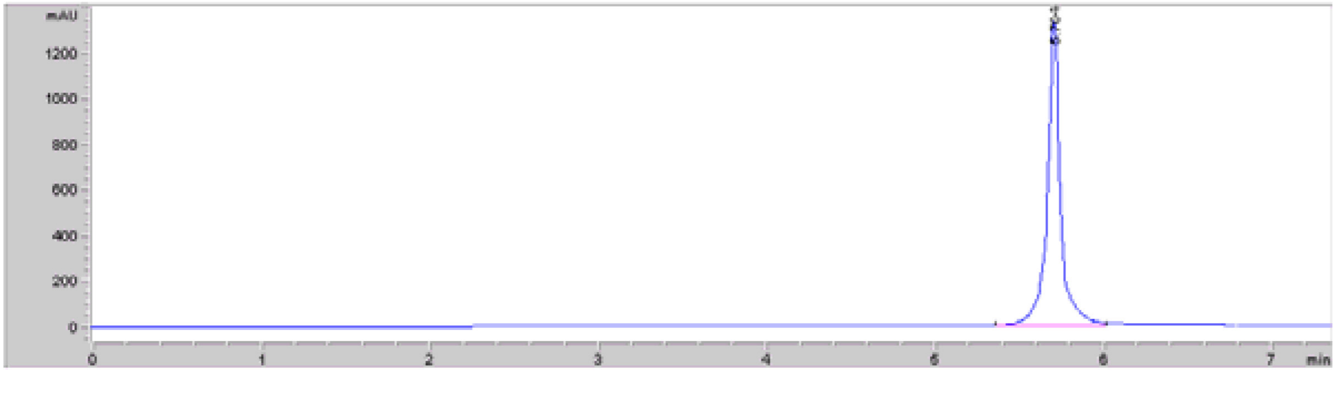


**Supplementary Figure 3: HPLC program of THPDTPI.**
